# Content validation and testing of a gamified web tool for nursing supervision

**DOI:** 10.1177/17449871251392436

**Published:** 2026-01-09

**Authors:** Raquel Silva, Rafael Camelo, Cristina Pinto, Maria Joana Campos, Marta Campos Ferreira, Carla Sílvia Fernandes

**Affiliations:** Alto Ave Local Health Unit, EPE, Portugal; Faculty of Engineering of the University of Porto, Portugal; Porto Higher School of Nursing, Porto, Portugal; Porto Higher School of Nursing, Porto, Portugal; INESC TEC – Institute of Systems and Computer Engineering, Faculty of Engineering of the University of Porto, Porto, Portugal; School of Health – Polytechnic Institute of Viana do Castelo; Aditgames – Association for Innovation, Technologies and Games in Health; Association ADITGames

**Keywords:** clinical supervision, educational technology, gamification, nursing education, nursing students

## Abstract

**Background:**

This study aimed to validate the content of a game focused on clinical supervision in nursing, with the collaboration of experts, and to assess its usability alongside a group of nurses. The development of SUPERVISE^®^ was grounded in theories of Experiential Learning, Self-Determination, Constructivist, and Social Cognitive.

**Methods:**

A mixed study design was used. In the first phase, the content of the game was validated with the participation of experts using a modified e-Delphi method. In the second phase, the usability of SUPERVISE^®^ was tested with nurses.

**Results:**

In the first phase, the content of the game was validated by 36 experts, reaching a consensus **=** 95.4% on the 128 questions on which the game was based. In the second phase, the SUPERVISE^®^ game was tested and evaluated by 39 nurses. It showed good usability and with a System Usability Scale score = 79.4 (above the cut-off of 68) and was recognised as an effective teaching strategy.

**Conclusion:**

This study highlights the importance of combining rigorous content validation with practical evaluation to develop effective gamified educational tools for nursing practice.

## Introduction

Practical training in clinical settings plays an important role in nurses’ education, enabling the development of clinical skills (Esteves et al., 2019; Immonen et al., 2019; Zhang et al., 2022). However, the effectiveness of this practical training depends significantly on the quality of the clinical supervision received by students, which directly influences the expertise and confidence of future nurses (Najafi Kalyani et al., 2019; Rodríguez-García et al., 2021; Voldbjerg et al., 2022). Clinical supervision is a formal process of professional support during clinical teaching, which contributes to the development of students’ technical and behavioural skills (Pramila-Savukoski et al., 2020). The whole process of clinical supervision encompasses reflection on the development of practice guided by qualified professionals, aiding in the socialisation and adaptation to newly qualified nurses (Almeida et al., 2024).

Although higher education institutions have a significant responsibility in the training of future nurses, professionals who act as supervisors in clinical practice play a key role in effectively promoting students’ clinical learning (Cant et al., 2021). Nurse supervisors, often referred to as mentors or facilitators, are responsible for creating a safe, positive, and encouraging learning environment in which students feel supported and motivated to reflect on their practice (Voldbjerg et al., 2022). The ability to establish positive relationships between supervisors and students is fundamental to strengthen the self-confidence and self-esteem of future professionals, giving them greater confidence in clinical decision-making (Anggeria & Damanik, 2022; Bifarin & Stonehouse, 2017).

Despite its importance, clinical supervision faces significant challenges that can compromise its effectiveness, including supervisors’ lack of time, work overload, and difficulties in providing constructive feedback to students. These are all factors that limit mentors’ availability to offer adequate guidance and support (Pramila-Savukoski et al., 2020). Although nurses possess clinical skills, mentoring students requires additional knowledge and skills, making it essential to implement specific training programmes for this role (Almeida et al., 2024; Dunlap and Fitzpatrick, 2023; Harvey et al., 2020). To overcome these limitations, ongoing training for supervisors becomes essential to enable them to act as effective role models for students (Esteves et al., 2019). Despite technological advances, traditional teaching continues to face challenges in meeting training needs, so it is vital to use new educational resources, which is where gamification emerges as an innovative approach (Fernandes et al., 2019; Fijačko et al., 2020). By integrating game elements into teaching, such as points, levels, badges, leaderboards, and challenges, gamification turns learning into a more engaging, interactive, and motivating experience, providing better results and encouraging behavioural changes (Fernandes et al., 2019, 2024a). The use of gamification elements in teaching has been widely explored in the healthcare system and is also applied both in academic training and in the continuous professional development of nurses (Almeida et al., 2024; Fernandes et al., 2018, 2024b).

Gamification has emerged as an innovative and engaging approach that is gaining popularity, particularly in nursing education (Almeida et al., 2024; Fernandes et al., 2018, Fernandes et al., 2019; Min et al., 2022; Gauthier et al., 2019). This teaching strategy is considered highly effective, as it captures the attention of individuals and increases their motivation for learning, thus promoting active involvement in the training process (Fijačko et al., 2020; McDougall, 2018). In addition, gamification has shown benefits in improving knowledge acquisition, decision-making, developing practical skills, and increasing participants’ self-confidence and self-efficacy (Sanz-Martos et al., 2024).

However, gamification is not limited to the educational field. Recent studies demonstrate its relevance in areas such as knowledge management, experience documentation, and organisational innovation. For example, Aghaei Mirakabad et al. (2024) conducted a scientometric study that mapped the role of gamification in knowledge management based on publications indexed in the Web of Science, highlighting its potential to increase motivation, knowledge sharing, and organisational efficiency.

The incorporation of innovative methods in supervision training can significantly improve the clinical guidance of nursing students (Almeida et al., 2024). However, the development of gamified resources requires a rigorous method, focused on the clear definition of objectives, including rules, collaboration, competition, and the integration of authenticated concepts into the game (Almeida et al., 2024; Fernandes et al., 2018). The development and evaluation of SUPERVISE^®^ were explicitly grounded in four complementary theoretical perspectives that support the professional development of nurse. Kolb’s Experiential Learning Theory (2015) provided the basis for structuring scenarios that enable supervisors to experience, reflect, conceptualise, and apply supervisory strategies. Self-Determination Theory (Ryan and Deci, 2020) guided the inclusion of motivational elements that promote autonomy, competence and relatedness in professional learning. Constructivist approaches (Piaget, 1973; Vygotsky, 1978) emphasised the active construction of supervisory knowledge through authentic challenges, ensuring a practice-centred process. Finally, Bandura’s Social Cognitive Theory (1986) highlighted the development of supervisory self-efficacy through observation, modelling, and reinforcement of behaviours. Together, these frameworks ensured that SUPERVISE^®^ was not merely a descriptive gamified tool but a theoretically informed educational intervention aligned with principles of adult learning and motivation. Faced with the challenges of clinical supervision and recognising the potential of gamification as an innovative strategy, this study aimed to validate the content of a game focused on clinical supervision in nursing and to assess its usability alongside a group of nurses. The study was guided by the following research questions: (1) Is the content of the SUPERVISE^®^ serious game considered valid and appropriate by experts in clinical supervision and nursing education? (2) What is nurses’ perception of the usability of the SUPERVISE^®^ serious game?

## Methods

### Study design

The study adopted a mixed study design, structured in two distinct phases. In the first phase, the content of the SUPERVISE game^®^ was validated by specialists in clinical supervision and nursing education using a modified e-Delphi method (Fernandes and Magalhães, 2024). This stage aimed to ensure the suitability and relevance of the game’s educational content by analysing the levels of consensus reached and the qualitative feedback provided by the experts. In the second phase, the educational usability of SUPERVISE^®^ was tested with the participation of nurses. The study was carried out between November 2023 and July 2024.

### E-Delphi

#### Expert selection

An exploratory study initially identified thematic areas for the game’s content, which was validated by experts using a modified e-Delphi method. Experts were recruited using purposive and snowball sampling. Inclusion criteria included postgraduate education or recognised expertise in clinical supervision, professional experience in nursing education and availability to participate in all Delphi rounds. In total, 36 experts agreed to participate. This method measures the reliability of content through consensus percentage and question stability, as outlined by Fernandes and Magalhães (2024) and Bownes and Giannotti (2023).

#### Development of the questionnaire

The initial questionnaire consisted of 128 items distributed across six thematic categories: (1) clinical supervision function, (2) clinical context, (3) clinical supervision strategies, (4) clinical supervisor, (5) supervisory relationship, and (6) clinical scenarios. These items were derived from the curricula of Portuguese nursing schools and supported by a review of the literature.

#### Delphi rounds

Round 1: Experts analysed each item and suggested reformulations, additions, or deletions where necessary; Round 2: These proposals were incorporated, and the new version was resubmitted until the previously established level of consensus was reached. Experts re-evaluated the revised items, and stability of responses was assessed.

#### Consensus and stability criteria

This method measures the reliability of content through consensus percentage and question stability, as outlined by Fernandes and Magalhães (2024) and Bownes and Giannotti (2023). In fact, in the literature on the Delphi technique, there are different levels of consensus, ranging from 50% to 97%, depending on the desired rigour and the context of the study (Fernandes and Magalhães, 2024). However, to ensure greater methodological robustness and reduce the possibility of including items with limited agreement, in this study, we established the criterion that each item would only be included in the game if it reached ⩾ 85% consensus and presented stability, defined as the absence of reformulation suggestions (Bownes and Giannotti, 2023; Fernandes and Magalhães, 2024). Questions were included in the game if they met an 85% consensus criterion and showed stability, indicated by a lack of alternative suggestions.

#### Outcome of the Delphi process

After two rounds, consensus was reached on 95.4% of the items, confirming the robustness of the content validation process and ensuring that the material included in the SUPERVISE^®^ game was considered relevant and adequate for training in clinical supervision.

#### Usability test

The usability of SUPERVISE^®^ was tested in a second phase using a mixed-method approach. This test aimed to evaluate nurse interactions with the game and its effectiveness in a clinical context, employing standardised scales to measure usability and educational impact.

### Participants

E-Delphi phase: Experts were selected through purposive and non-probability snowball sampling and contacted via email to access a Microsoft Forms^®^ platform. Microsoft Forms® platform (Microsoft Corporation, Redmond, WA, USA), a secure web-based tool included in the Microsoft 365 suite, which complies with GDPR standards for data protection. Eligible participants included nurses with significant clinical supervision skills, postgraduate education, or research experience in clinical supervision. The panel comprised 36 experts.

Usability test: Similarly, 39 nurses were selected via non-probability snowball sampling and invited by email to assess the game on Microsoft Forms^®^. Nurses from various professional backgrounds participated, regardless of their specific training in clinical supervision.

### Instruments and data collection

E-Delphi phase: The initial set of 128 true/false and multiple-choice questions was developed from scratch, based on the syllabuses of Nursing Supervision courses taught at various Nursing Schools in Portugal. These questions were subsequently organised into six thematic categories: clinical supervision function, clinical context, clinical supervision strategies, clinical supervisor, supervisory relationship, and clinical scenarios. In the first round of the e-Delphi process, experts analysed each item and suggested reformulations, additions or deletions where necessary. These proposals were incorporated, and the new version was resubmitted in the second round until the previously established level of consensus was reached. Data collection occurred from November 2023 to January 2024. Usability test: A similar two-part questionnaire was used for the usability test, where the first part gathered participant demographics and professional backgrounds, and the second part evaluated the game. This evaluation used two scales: the Game Usability Scale (Portuguese version of the System Usability Scale [SUS]) and the Serious Educational Game in Nursing – Appraisal Scale (SEGiNAS), both employing a 5-point Likert scale to gauge agreement levels. Open-ended questions collected feedback on the game’s pros and cons. The SUS was used to assess usability. It consists of 10 items answered on a 5-point scale (1 = strongly disagree to 5 = strongly agree). The final score ranges from 0 to 100, with scores above 68 indicating above-average usability. This study applied the European Portuguese version, validated by Martins et al. (2015), which demonstrated construct validity confirmed by significant correlations with other usability measures.

To assess the educational perception of the game, SEGiNAS, developed and validated by Fernandes et al. (2024), was used. The scale contains 20 items in Likert format (1 = strongly disagree to 5 = strongly agree), distributed across three dimensions: Engagement and Teaching Effectiveness (8 items), Learning Impact and Practical Application (6 items) and Relevance and Clarity of Content (6 items). The sum of the items provides a total score, with higher values representing a better rating of the game. Data collection for this phase was between June and July 2024.

### Data analysis

In the modified e-Delphi study, data analysis was conducted using both quantitative and qualitative approaches. Quantitatively, a 3-point Likert scale assessed expert consensus on the content: 1 – Agree without changes, 2 – Agree with suggested changes and 3 – Disagree. Qualitatively, the experts’ suggestions were validated using Bardin’s (2016) content analysis, with categories generated inductively from the responses. Two researchers collaborated independently, subsequently discussing disagreements until consensus was reached, ensuring inter-rater reliability. Qualitatively, expert suggestions were analysed using Bardin’s content analysis methodology to refine the questions and answers. Similarly, data from the game’s usability testing were subjected to quantitative analysis of central tendency measures and qualitative content analysis, conducted according to Bardin’s (2016) stages of pre-analysis, exploration, treatment of results, inference, and interpretation.

### Ethical considerations

For this study, authorisation was requested and obtained from the ethics committee Fluxo CE_27/2023 and at all stages, the participants were informed about the purpose and objectives of the study, with the guarantee of anonymity and confidentiality of the data.

### Game SUPERVISE^®^

**Figure 1.**
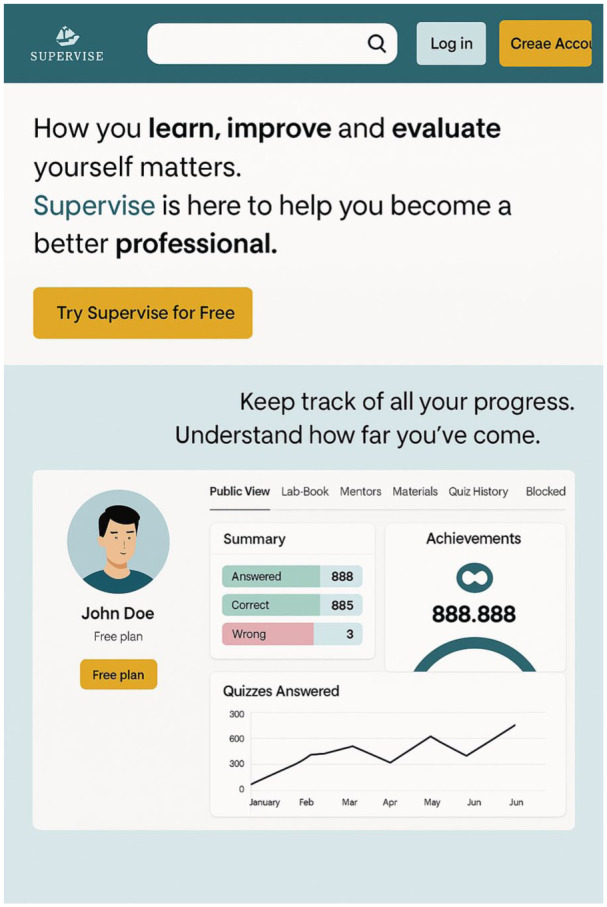
SUPERVISE® application, fullscreen interface.

SUPERVISE^®^ is a serious game designed for clinical supervision, aimed at supporting professional growth and enhancing reflective practice. It is accessible on both computers and mobile devices and facilitates interaction between supervisors and supervisees (students) (Figure 1). The game’s primary goal is to train supervisors to improve the effectiveness and interactivity of the supervision process. It supports two user profiles – supervisors and supervisees – and features various interactive tools to foster effective communication. Key features include the ‘Answer Quiz’ interface, which allows for questions with detailed answers and space for comments, and the ‘Profile’ interface, showcasing resources like ‘Logbook’ for recording experiences, ‘Supervisee’ for student communication and monitoring, and ‘Quiz History’. These elements collectively enhance the learning experience and were utilised in this study to validate the quiz content.

## Results

The presentation of the results will follow the same format as described in the methodology: Phase 1: validation of the game’s content using a modified e-Delphi method. Phase 2: usability testing of SUPERVISE^®^ with nurses.

### E-Delphi results

#### Expert characterisation: E-Delph round 1

In the first round of the e-Delphi study, 45 out of the 189 invited experts participated, corresponding to a response rate of 23.9%. The socio-demographic and professional characteristics of the experts are summarised in Table 1. Most participants were female (73.3%), with a predominance of highly qualified professionals: 35.6% held a master’s degree, 40.0% a doctoral degree, and 24.4% held a bachelor’s degree in nursing. Professional experience ranged from 6 to 42 years. Regarding their field of practice, 55.6% worked in hospitals, 40.0% in higher education and 4.4% in primary healthcare. Experience in clinical supervision ranged from 5 to 34 years, reflecting a participant profile with consolidated knowledge and practical expertise in the field.

**Table 1.** Socio-demographic and professional characterisation of participants.

Socio-demographic and professional characterisation	Modified E-Delphi				Game test	
	Round 1		Round 2			
	*N*	%	*N*	%	*N*	%
Gender						
Male	12	26.7	11	30.6	6	15.4
Female	33	73.3	25	69.4	33	84.6
Academic qualifications						
Bachelor’s degree	11	24.4	6	16.7	25	64.1
Master degree	16	35.6	17	47.2	12	30.8
PhD	18	40.0	13	36.1	2	5.1
Years of professional experience						
Minimum	6	–	6	–	2	–
Maximum	42	–	41	–	34	–
Professional activity						
Teaching	18	40.0	14	38.8	3	7.7
Nurse in the clinical context	25	55.6	20	55.6	35	89.7
Manager	2	4.4	2	5.6	1	2.6
Years of experience in clinical supervision						
Minimum	5	–	5	–	–	–
Maximum	34	–	34	–	–	–
Has training in clinical supervision in nursing?						
Yes	–	–	–	–	19	48.7
No	–	–	–	–	20	51.3
Have you had experience as a Clinical Supervisor?						
Yes	-	-	-	-	34	87.2
No	-	-	-	-	5	12.8

#### E-Delph results: round 1

From this stage, 128 questions and their answers were sent, categorised according to clinical supervision, clinical context, clinical supervision strategies, clinical supervisor, supervisory relationship, and clinical scenarios. The level of consensus reached for all the questions was 96%, with no need to eliminate any of them. The suggested changes only had an impact on the stability of 77 questions, which were submitted to the second round of the e-Delphi method.

#### Expert characterisation: E-Delph round 2

In the second round of the e-Delphi study, 77 questions were included, organised into six thematic categories. A total of 36 out of the 45 experts from the previous round participated (80%). Their characteristics are also shown in Table 1. The majority were female (69.4%) and held high academic qualifications (47.2% held a master’s degree and 36.1% a doctoral degree). Professional experience ranged from 6 to 41 years, and experience in clinical supervision from 5 to 34 years. Regarding professional context, 55.6% worked in hospitals, 38.8% in higher education, and 5.6% in primary healthcare.

#### E-Delph results: round 2

In the second round, all the changes made in the first round were accepted. The level of consensus obtained was 97.9%, which, together with the stability of the questions, supported the acceptance of all 77 questions submitted in the study, guaranteeing the stability of the content and not requiring any further rounds.

### Usability test results

#### Participants characterisation

A total of 39 nurses completed the evaluation of SUPERVISE^®^. Their demographic and professional characteristics are presented in Table 1. Following the use of SUPERVISE^®^, 39 nurses completed the evaluation questionnaire. The majority were female (84.6%) and held a bachelor’s degree in nursing (64.1%), whereas 30.8% had a master’s degree and 5.1% a doctoral degree. Professional experience ranged from 2 to 34 years, and 89.7% were engaged in clinical practice. Regarding clinical supervision training, 48.7% reported having received it. Among these, 87.2% had experience as clinical supervisors.

#### Usability tests results

Regarding the usability evaluation of SUPERVISE^®^, the participants provided positive responses, indicating that the prototype was easy to use, useful and efficient, although its functionality could be improved. This will make it possible to enhance the user experience in the future. The results of the SUS applied to SUPERVISE^®^ are described in Table 2. The means, standard deviations, and minimum and maximum values for each of the 10 items were presented. The overall SUS score, calculated from the individual responses, was 79.4 points, above the cut-off point of 68, considered indicative of good usability (Martins et al., 2015). The average response for items one, three, five, seven and nine was 4.22, which corresponds to the ‘Partially agree’ option on the Likert scale. The average response for items two, four, six, eight and ten was 1.87, which corresponds to the ‘Partially disagree’ option on the same scale. The average standard deviation was 0.99, indicating considerable diversity in the participants’ opinions, with some agreeing or disagreeing more strongly than others in relation to the prototype. Regarding the evaluation of SUPERVISE^®^ as a teaching strategy for nurses, the results are shown in Table 3. The average response was 4.2 (‘Partially agree’ on the Likert scale), with an average standard deviation of 0.96, indicating that the participants expressed a predominantly positive opinion, although not total agreement.

**Table 2.** Results of the usability evaluation of ‘SUPERVISE’ with SUS.

SUS	Mean (SD)	Min–Max
1. I think that I would like to use this system frequently.	3.92 (0.89)	1–5
2. I found the system unnecessarily complex.	2.38(1.27)	1–5
3. I thought the system was easy to use.	4.38 (0.84)	1–5
4. I think that I would need the support of a technical person to be able to use this system.	1.69 (1.04)	1–4
5. I found the various functions in this system were well integrated.	4.21 (0.91)	2–5
6. I thought there was too much inconsistency in this system.	1.92 (1.00)	1–4
7. I would imagine that most people would learn to use this system very quickly.	4.44 (0.74)	2–5
8. I found the system very cumbersome to use.	1.59 (1.01)	1–5
9. I felt very confident using the system.	4.15 (1.00)	1–5
10. I needed to learn a lot of things before I could get going with this system.	1.77 (1.21)	1–5
Overall SUS score (0–100)	79.4	–

SUS: System Usability Scale.

**Table 3.** Results of the evaluation of SUPERVISE^®^ as a strategy in teaching nurses with SEGiNAS.

Serious Educational Game in Nursing – Appraisal Scale (SEGiNAS)	1: Strongly disagree		2: Partially disagreed		3: Neither agree nor disagreed		4: Partially agree		5: Strongly agree		Mean	Mode	Standard deviation
	*n*	%	*n*	%	*N*	%	*n*	%	*n*	%			
1. The use of this serious game contributes to a better understanding of the educational material.	1	3%	3	8	5	13	12	31	18	46	4,10	5	1,06
2. Considers the content covered in this serious game to be useful for the development of the professional activity.	1	3	2	5	6	15	16	41	14	36	4,03	4	0,97
3. The time allocated to the application of this serious game is adequate.	4	10	4	10	9	23	14	36	8	21	3,46	4	1,22
4. The themes of this serious game are presented clearly and coherently.	2	5	2	5	1	3	12	31	22	56	4,28	5	1,08
5. The content of this serious game is appropriate between theory and practice.	1	3	0	0	3	8	13	33	22	56	4,41	5	0,84
6. You believe that learning from this serious game will have an impact on your performance in practice.	1	3	0	0	7	18	18	46	13	33	4,08	4	0,86
7. The use of this serious game contributes to the motivation of the trainees.	1	3	0	0	8	21	14	36	16	41	4,13	5	0,91
8. You felt that your knowledge of the area had evolved with the use of this serious game.	2	5	1	3	9	23	14	36	13	33	3,90	4	1,06
9. You intend to apply the knowledge acquired with this serious game.	2	5	0	0	6	15	13	33	18	46	4,15	5	1,03
10. The amount of content developed in this serious game was appropriate to your level of knowledge.	2	5	2	5	5	13	17	44	13	33	3,95	4	1,06
11. The opportunity provided by this serious game encouraged the participation of the trainees.	2	5	1	3	9	23	17	44	10	26	3,82	4	1,01
12. You think this strategy is useful for learning.	1	3	1	3	3	8	16	41	18	46	4,26	5	0,90
13. The duration of this serious game is adequate for learning.	1	3	3	8	8	21	17	44	10	26	3,82	4	0,98
14. You would recommend the content covered in this serious game to others.	1	3	1	3	2	5	16	41	19	49	4,31	5	0,88
15. This serious game is useful for reviewing knowledge.	1	3	0	0	2	5	10	26	26	67	4,54	5	0,81
16. The use of this serious game is a good resource for sparking interest in the subject.	1	3	0	0	3	8	14	36	21	54	4,38	5	0,84
17. This serious game is useful for identifying knowledge gaps.	1	3	0	0	5	13	18	46	15	38	4,18	4	0,84
18. This serious game helps to retain specific knowledge.	1	3	2	5	3	8	12	31	21	54	4,28	5	0,99
19. This serious game is a suitable teaching strategy for acquiring skills.	1	3	1	3	6	15	15	38	16	41	4,13	5	0,94
20. Overall, the use of this serious game was satisfactory for me.	1	3	1	3	3	8	15	38	19	49	4,28	5	0,90

**Figure 2.**
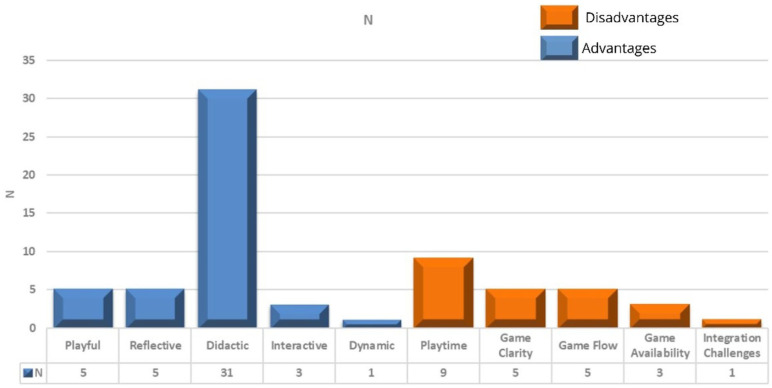
Advantages and disadvantages of SUPERVISE.^®^

In the open questions about the advantages and disadvantages of the game, a content analysis was carried out, as shown in Figure 2. For the advantages, five subcategories were identified: playful, reflective, didactic, interactive, and dynamic. Five subcategories expressing the disadvantages of the game were also identified: the duration of the game, the understanding of it, its dynamics, its accessibility and, finally, the difficulty of putting the content into practice. In addition, 15 of the 39 participants said they had not identified any disadvantages, reinforcing the overall positive perception of SUPERVISE^®^ as an innovative teaching strategy.

## Discussion

This study allowed us to validate the content of a game called SUPERVISE^®^, designed to improve the clinical supervision process for nursing students and to assess its usability. The theoretical foundation of SUPERVISE^®^ represents a key strength of this study. Grounding the tool in Kolb’s Experiential Learning Theory (1984, 2015) allowed us to structure interactive scenarios that mirror the experiential-reflective cycle fundamental to supervisory practice. Self-Determination Theory (Ryan and Deci, 2020) provided a framework for designing features that sustain motivation, such as autonomy in decision-making, perceived competence through feedback, and identification with the supervisory role. Constructivist approaches (Piaget, 1973; Vygotsky, 1978) reinforced the idea that supervisors actively build knowledge when faced with authentic clinical challenges, whereas Bandura’s Social Cognitive Theory (1986) highlighted the importance of enhancing supervisory self-efficacy through repeated practice, modelling, and reinforcement. By integrating these perspectives, SUPERVISE^®^ supports not only the acquisition of supervisory knowledge but also motivation and reflective practice, positioning it positive feedback as a theoretically informed intervention for the professional development of nurses.

The clinical supervision process is an essential pillar in the training of future nurses, playing a decisive role in the development of technical and behavioural skills (Immonen et al., 2019; Pramila-Savukoski et al., 2020). However, the challenges faced by clinical supervisors, namely the need for specific training, compromise the effectiveness of this process and highlight the need for new educational approaches to train these professionals (Pramila-Savukoski et al., 2020). Acquiring knowledge about the clinical supervision process is fundamental to ensuring that students receive structured, quality supervision, which requires effective educational strategies and innovative resources (Almeida et al., 2024). Studies have shown that serious games can improve knowledge acquisition, information retention, and skills development, making the training process more effective and engaging (Fernandes et al., 2024a; Sanz-Martos et al., 2024).

In the case of SUPERVISE^®^, the game was designed to meet the specific needs of clinical supervisors and students, combining playful, reflective, and interactive elements to provide an enriching learning experience in line with the demands of professional practice. However, the implementation of new educational resources requires a rigorous validation process before use (Fernandes et al., 2018). This study followed this approach by structuring itself into two complementary phases: the first, dedicated to validating the content with experts, and the second, focused on evaluating the usability and educational perception of the game among nurses. Content validation using the modified e-Delphi method ensured that the topics covered in the game were relevant and suitable for clinical supervision training. The modified e-Delphi study made it possible to robustly validate the content of the game, guaranteeing its relevance and academic rigor. This methodological approach has been widely recognised as an effective strategy for gathering consensus among experts and improving the quality of educational content (Bownes and Giannotti, 2023; Fernandes and Magalhães, 2024). The high level of consensus obtained among the experts (95.4) and the need for only two rounds reinforces the credibility and applicability of the content developed.

At the same time, the usability evaluation alongside nurses made it possible to test the viability and acceptance of the tool, providing essential feedback for possible improvements before its widespread implementation. In general, participants gave SUPERVISE^®^ positive feedback, indicating that it is usable and effective as a teaching strategy for nurses. The overall value of the SUS was 79.4 points, above the cut-off point of 68, considered indicative of good usability (Martins et al., 2015). These confirm that SUPERVISE^®^ presents good usability, reinforcing its suitability as an educational tool for clinical supervision in nursing. This recognition is in line with the literature, which identifies gamified elements as an effective strategy for engaging users, promoting interest, motivation, knowledge acquisition, and learning (Almeida et al., 2024; Sarker et al., 2021). In addition they favour knowledge retention (Siqueira et al., 2020), and are a valuable resource for reviewing and consolidating acquired knowledge (Ravyse et al., 2017) improving not only theoretical knowledge but also practical skills (Min et al., 2022). In addition to overall usability, it is important to highlight the gamification mechanics embedded in SUPERVISE^®^, as these contribute directly to user engagement and educational effectiveness. The game incorporates scenario-based challenges in which supervisors are required to analyse clinical situations and choose the most appropriate supervisory actions, fostering critical thinking and reflective practice. A reward system based on points and immediate performance feedback provides positive reinforcement, enhancing perceived competence and sustaining motivation. Furthermore, the game includes adaptive features, such as progressive levels of difficulty and varied clinical contexts, which allow supervisors to gradually consolidate skills and ensure relevance across different levels of supervisory experience. These mechanisms not only enhance the learning experience but also strengthen the replicability of the intervention by making explicit the structural elements that underpin its educational impact. In the case of SUPERVISE^®^, the multifunctional design offers an educational experience that is both engaging and informative, aligned with the needs of supervisors and supervisees (students). In the usability phase of the resource and perception of its use, among the 39 participants, 15 did not identify any disadvantages, which reinforces the overall positive perception of SUPERVISE^®^ as an innovative teaching strategy. These results are in line with the findings of Almeida et al. (2024), who also found a favourable reception to the use of a board game on clinical supervision in nursing. Nevertheless, participants also highlighted specific aspects of the game’s functionality that could be improved. The most frequently mentioned limitations included play time, clarity of instructions, and accessibility. These factors may influence the scalability and widespread adoption of SUPERVISE^®^ in clinical and educational contexts. We acknowledge these criticisms as important feedback and have framed them as opportunities for iterative refinement in future versions of the game.

The implications of this study extend beyond the validation of SUPERVISE^®^ as an educational tool, situating it within broader academic and policy-oriented frameworks. International guidelines and models developed in countries such as the United Kingdom, Canada, and Australia have long emphasised the importance of structured clinical supervision to enhance care quality and student learning outcomes (DeCicco, 2008). At the same time, global policy discussions highlight the integration of digital education, gamification and competency-based approaches as key strategies to prepare health professionals for the demands of contemporary healthcare. Within this context, SUPERVISE^®^ emerges as an empirically grounded innovation that can be scaled and embedded in both preregistration nursing curricula – supporting the integration of theory into practice, and structured continuing professional development (CPD) programmes, sustaining reflective practice and supervisory competence among registered nurses. These links underscore the potential of SUPERVISE^®^ not only as a pedagogical innovation but also as a resource that aligns with international policy priorities in nursing education and workforce development. Nevertheless, although SUPERVISE^®^ is accessible on both mobile and desktop platforms, potential challenges related to digital literacy and access disparities must be acknowledged. Although the game was designed to be intuitive and user-friendly, variations in digital proficiency among nurses may influence its uptake. In addition, unequal access to technological infrastructure – particularly in lower-resource clinical environments – may limit the universal applicability of the game. These contextual factors should be considered when interpreting the findings, as they may restrict generalisability. Future research should therefore examine the feasibility and usability of SUPERVISE^®^ in diverse healthcare settings and explore strategies to ensure equitable access and effective use.

In addition to the need for longitudinal research, it is also important to consider the ecological validity of SUPERVISE^®^. Future studies should therefore include pilot implementations in real-time clinical supervision contexts, allowing the tool to be tested under authentic conditions of practice. Such studies would provide valuable insights into the feasibility, acceptability, and educational impact of SUPERVISE^®^.

### Limitations

Despite its contributions, this study has limitations. The sample size, particularly in the usability test phase with nurses, is small, which might limit the generalisation of the findings to broader clinical and educational settings. A larger and more diverse sample could enhance the applicability of SUPERVISE^®^. In addition, the absence of a control group limits the ability to draw causal inferences regarding the educational impact of SUPERVISE^®^, highlighting the need for comparative studies with alternative teaching strategies. Participation was voluntary, which introduces the possibility of self-selection bias, as nurses with greater interest in innovative tools may have been more likely to participate. Finally, the assessment was conducted in the short term and focused primarily on usability and immediate perceptions. Future longitudinal studies are required to evaluate the sustained impact of SUPERVISE^®^ on supervisory skills and educational outcomes over time. Additionally, the study assessed only the short-term usability and educational impact of the game, without considering its long-term effects on supervisors and students. Additionally, the use of purposive and snowball sampling strategies may have introduced selection bias, as participants were often recruited within shared professional or academic networks. This could have limited the representativeness of the sample and potentially influenced the perspectives collected, particularly regarding attitudes towards digital tools and gamification. Moreover, participants’ prior experience with digital tools and gamification was not systematically assessed. It is possible that familiarity with digital platforms may have influenced their perceptions of usability, which should be considered in future studies. Therefore, caution is needed when generalising the findings to broader nursing populations. Nonetheless, this research marks a significant advancement in using serious games for training clinical supervisors, offering a validated and tested model for further development and research.

## Conclusion

Clinical supervision plays a fundamental role in the training of nursing students and requires innovative strategies to improve the quality of the care provided. In this context, the SUPERVISE^®^ game, whose content was validated by experts and then tested by nurses, proved to be an innovative and effective tool, combining interactive, reflective, and didactic elements to optimise the supervision process in clinical settings. Content validation using the modified e-Delphi method ensured that the topics covered were relevant and appropriate. In addition, the usability and overall positive perception of the participants, including the absence of disadvantages mentioned by a significant number of nurses, reinforces the feasibility of its implementation in educational practice. However, future studies should evaluate the effectiveness of SUPERVISE^®^ in different clinical contexts, and there is a need for longitudinal studies that analyse the medium- and long-term impact of its use in nursing education, including its influence on the clinical context and professional development. SUPERVISE^®^ also aligns with international policy priorities in nursing education. Its potential integration into pre-registration curricula and Continuing Practice Development (CPD) programmes highlights its relevance not only as an innovative educational tool but also as a resource that can support broader strategies to strengthen clinical supervision and competency-based training.

This work aligns with current educational and professional policy frameworks that advocate for innovative, evidence-based approaches to enhance the quality of nursing supervision and education. By providing a rigorously validated and tested digital tool, SUPERVISE^®^ can inform policy development in areas such as clinical training standards, integration of digital pedagogical resources, and CPD of nurse supervisors.

**Key points for policy, practice and/or research**

- The gamified web tool SUPERVISE^®^ demonstrated high usability and was perceived as a valid teaching strategy for integration into clinical nursing supervision.
- Future research should evaluate the medium- and long-term impact of gamified elements and web tools for nursing supervision, such as SUPERVISE^®^, on the supervision process for both supervisors and students over time.
- The integration of gamification elements into the clinical supervision process in nursing may increase engagement, motivation, and reflective practice among nursing students, supporting skills development and knowledge retention.
- These results support the potential of serious games to inform the development of innovative, evidence-based educational policies for nursing education and professional development.
